# Nasopharyngeal carriage of *Streptococcus pneumoniae* among healthy children in Kassena-Nankana districts of Northern Ghana

**DOI:** 10.1186/s12879-021-06302-5

**Published:** 2021-07-08

**Authors:** Deborah K. Narwortey, Alex Owusu-Ofori, Hans-Christian Slotved, Eric S. Donkor, Patrick O. Ansah, Paul Welaga, Godfred Agongo, Abraham R. Oduro

**Affiliations:** 1 Navrongo Health Research Centre, Ghana Health Services, Biomedical Department, P.O. Box 114, Navrongo, Ghana; 2 Department of Clinical Microbiology, School of Medicine and Dentistry, Kwame Nkrumah University of Science and Technology, Kumasi, Ghana; 3 Department of Bacteria, Parasites and Fungi, Statens Serum Institut, Copenhagen, Denmark; 4 Department of Medical Microbiology, University of Ghana Medical School, Accra, Ghana

**Keywords:** *S. pneumoniae*, PCV-13, Carriage, Vaccine serotypes, Non-vaccine serotypes, Northern Ghana

## Abstract

**Background:**

Pneumococcal vaccine immunizations may be responsible for alterations in serotype epidemiology within a region. This study investigated the pneumococcal carriage prevalence and the impact of the 13-valent pneumococcal conjugate vaccine (PCV-13) on circulating serotypes among healthy children in Northern Ghana.

**Methods:**

This was a cross sectional study conducted in the Kassena-Nankana districts of Northern Ghana from November to December during the dry season of 2018. Nasopharyngeal swabs collected from 193 participants were cultured per standard microbiological protocols and pneumococcal isolates were serotyped using the latex agglutination technique and the capsular Quellung reaction test. We examined for any association between the demographic characteristics of study participants and pneumococcal carriage using chi-square test and logistic regression.

**Results:**

Of the 193 participants that were enrolled the mean age was 8.6 years and 54.4% were females. The carriage rate among the participants was 32.6% (63/193), and twenty different serotypes were identified. These included both vaccine serotypes (VT), 35% (7/20) and non-vaccine serotypes (NVT), 65% (13/20). The predominant serotypes (34 and 11A), both of which were NVT, accounted for a prevalence of 12.8%. PCV-13 covered only 35% of serotypes identified whiles 40% of serotypes are covered by PPV 23.

**Conclusion:**

Post-vaccination carriage of *S. pneumoniae* is high and is dominated by non-vaccine serotypes. There is therefore a need for the conduct of invasive pneumococcal disease surveillance (IPD) to find out if the high non-vaccine serotype carriage translates to disease. And in addition, a review of the currently used PCV-13 vaccine in the country would be considered relevant.

**Supplementary Information:**

The online version contains supplementary material available at 10.1186/s12879-021-06302-5.

## Background

*Streptococcus pneumoniae,* is described as a pathobiont inhabiting the host nasopharynx as a commensal and causes several invasive and non-invasive diseases [1, 2]. High proportions of disease incidence have been associated with the pneumococcus in recent times, contributing to high levels of disease burden globally. Annually, an average of 800,000 deaths are reported among children due to invasive pneumococcal diseases with considerable proportions occurring in developing countries [3, 4]. Globally an estimated 50% of all pneumococcal deaths reported in 2015 occurred in developing countries [5].

Nasopharyngeal carriage of the pneumococcus may ultimately lead to severe disease conditions including septicaemia, meningitis, pneumonia, sinusitis and otitis media [6]. The pneumococcus exhibits a nonrandomized serotype distribution within the carriage reservoir and this is determined by variations in age, time and geographical jurisdictions [3]. Currently, up to 100 pneumococcal serotypes has been described [7], from which an average of 20 serotypes are reported to be commonly associated with juvenal invasive pneumococcal disease (IPD), namely 19A, 3, 7F, 23F, 11, 6A, 6B, 14, 8, 18C and 19F [2, 8].

Since the introduction of vaccines against pneumococcus, substantial health benefits have been realized despite suggestions of serotype replacement following implementation of these vaccines [9, 10]. Currently two main forms of pneumococcal vaccines are in existence namely pneumococcal polysaccharide vaccines (PPVs) which targets 23 antigenic types of the pneumococcus and pneumococcal conjugate vaccines (PCVs) which targets 10 and 13 antigenic serotypes [11–13]. Whereas PCVs offer better protection for children, eliciting herd protection, inducing effective immune bodies which are long lived, PPVs are known to confer protection specifically for the adult population and immune compromised individuals [14, 15]. Pneumococcal conjugate vaccines, though effective in inducing immune response across all age groups, cover a limited number of serotypes [16, 17].

Ghana introduced the 13-valent conjugate vaccines (PCV-13) into its Expanded Programme for Immunization (EPI) in 2012 for infant vaccinations at 6, 10 and 14 weeks of age [18]. Nonetheless, the targeted serotype range of the vaccine is quite limited. A previous study from Ghana prior to the introduction of the vaccine in 2012 indicated a vaccine serotype coverage of approximately 50% [19]. Limitation in serotype coverage could exert a vaccine selective pressure inducing evolution of non-vaccine serotypes [20]. Thus, a post vaccination upsurge in non-vaccine type serotypes (NVT) is likely to favour transmissions and emergence of NVT pneumococcal attacks.

Serotype replacement post pneumococcal vaccination deployment is thus a major issue of concern and may pose significant health challenges. This highlights the need for maintaining carriage surveillance in order to explore the dynamics in serotype distribution within the carriage reservoir and other post-vaccination impacts [21]. In Ghana, several post-vaccination pneumococcal surveillance studies have been carried out [22–24]. However, these studies were limited to southern Ghana and there is limit pneumococcal post-vaccination surveillance in the northern part of the country where pneumococcal outbreaks are relatively common. This study aimed at investigating the rate of nasopharyngeal carriage of *S. pneumoniae* and the serotypes circulating among school-aged children aged 5 to 12 years in the Kassena-Nankana districts of Ghana.

## Methods

### Study site

**Fig. 1:**
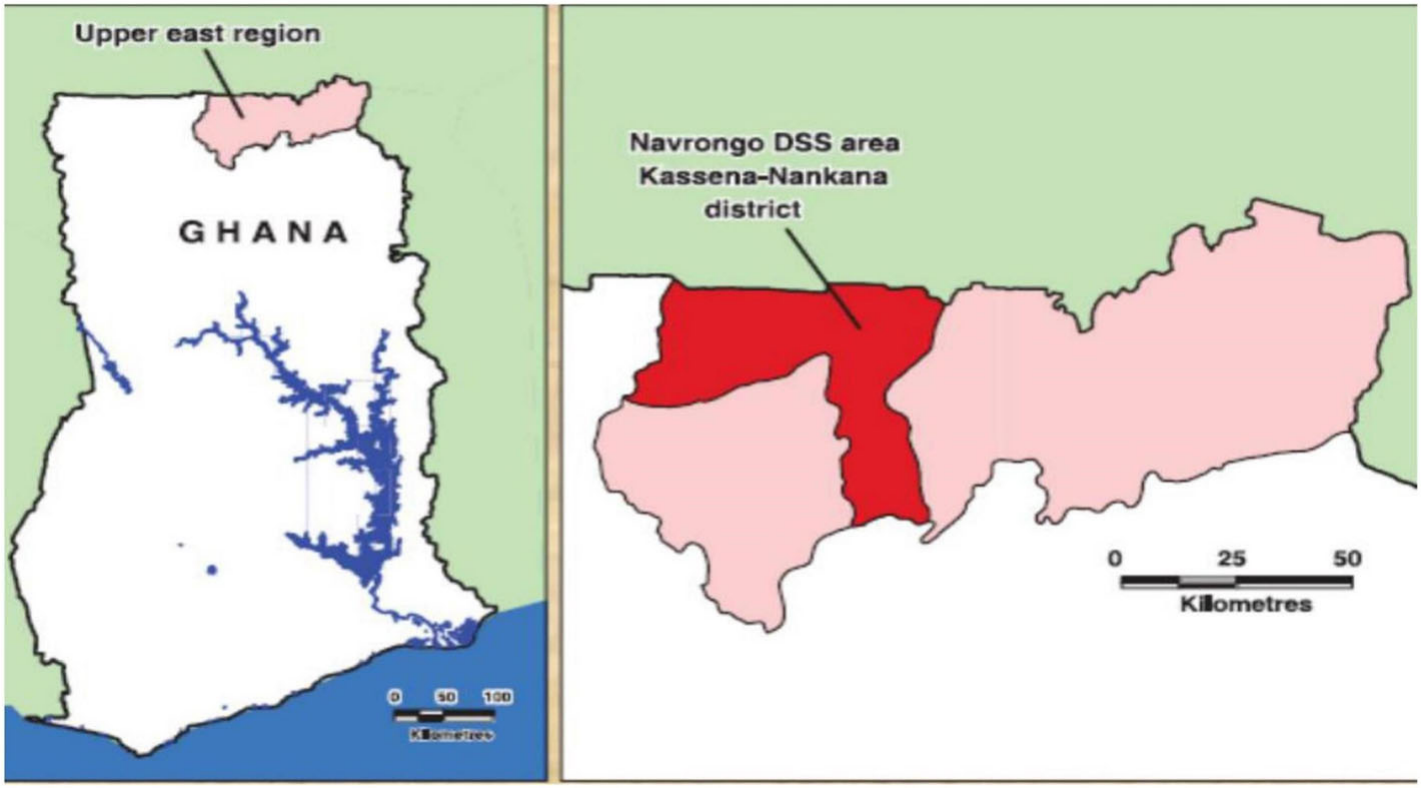
Map Of Ghana Showing the Upper East Region in Left Panel and Map of the Upper East Region Showing The KND Area in Right Panel. Sauce: (Oduro et al. 2012)

The study was conducted in the Kassena–Nankana Districts (KND) of Northern Ghana, which is predominantly characterized by arid climatic conditions with a population of 165,000 and population density of 91.5km^2^ [25]. The study area has a Health and Demographic Surveillance System (HDSS) which monitors the population dynamics of the area [25]. Fieldworkers visit all the households 2–3 times a year and register all demographic events such as births, deaths, marriages, in-migrations and out-migrations. The district has a rural setting except for those living in the town of Navrongo, which has a population of 20,000 with an average of 10 inhabitants living in a compound [26] (Fig. 1). The study area lies within the meningitis belt of sub-Saharan Africa where there are frequent outbreaks of meningitis during the dry seasons (between December and May) with seasonal occurrence of larger outbreaks reported every 8–12 years [27, 28]. In 2005, the district reported a serotype 1 pneumococcal meningitis outbreak resulting in a case fatality rate of 44.4% out of the 117 pneumococcal meningitis cases recorded during the outbreak season [26]. The current coverage of PCV-13 vaccination in Ghana is reportedly 97% [29].

### Study design and data collection

This was a cross-sectional study carried out from November to December 2018 during the dry season. The study population involved children of both sexes, aged between 5 and 12 years. A multi-staged random sampling technique was adopted. First, we randomly sampled four communities within the study districts. In the second stage, we randomly sampled 50 eligible children comprising healthy children between 5 to 12 years whose parents have granted consent for participation from the communities using the database of the Navrongo HDSS which monitors the population dynamics of the area. Community engagement was undertaken to sensitize the selected communities on the aims and objectives of the project, all sampled children were then contacted through their parent to be part of the study. During data collection each household was visited, introductions were made, consent obtained. Data collection involved a four-member team comprising the investigator, field assistant, field worker and a consenter. Invitations to all selected participants were carried out a day prior to recruitments by the designated field worker and a parental informed consent process was conducted by a field consenter who ensured that parents were well informed about the survey.

A pre-piloted questionnaire (Supplemental file 1) was used to collect data on the demographic factors such as age, sex, and house-hold size as well as medical history and PCV-13 vaccination status of participants.

### Sample size estimation

Sample size was determined using the sample size formula N = Z_α_^2^ × P (1-P)/d2 as cited elsewhere [30]. With the assumption that the prevalence of pneumococcal carriage was 15% [31], a 95% confidence level and an accuracy rate of 5%, the sample size was estimated to be 196 participants. This was adjusted for a 5 % non-response rate resulting in a total sample size of 206 participants.

### Specimen collection and processing

Nasopharyngeal specimens were collected according to the WHO working group standard method [32] for detecting carriage of *S. pneumoniae*. In brief, nylon flocked swabs were used to collect samples from participants and transported in skimmed milk tryptone glucose glycerol (STGG) transport medium under a cold chain to the lab within six hours after collection [33].

Samples were cultured on 5 mg/L gentamicin sheep blood agar and incubated at 37 °C for 24 h under 5% CO_2_ atmospheric conditions. A well isolated pure colony showing alpha hemolysis was subcultured on blood agar and gram stained. Biochemical testing comprising bile solubility testing and optochin susceptibility testing were used to confirm suspected isolates [33]. Pneumococcal isolates were stored in 10% glycerol Brain-Heart Infusion (BHI) broth and subsequently shipped on dry ice to Statens Serum Institut (SSI), Copenhagen, Denmark.

The serotyping of the isolates was perform as described by Slotved and colleagues [34]. Briefly, a latex agglutination test was performed by using the ImmuLex™ test (ImmuLex™ Pneumotest kit, SSI Diagnostica, Denmark). Ten microliters of an overnight culture in Todd Hewit broth (TH-broth, SSIDiagnostica, Denmark) was reacted with serotype specific antisera for observation of agglutination reactions and confirmed by the Quellung reaction using serotype specific antisera (SSIDiagnostica, Denmark) [34, 35]. Non-typeable strains were defined as isolates presenting no phenotypic detectable capsule [23].

### Data analysis

Data was double entered and verified using EPI data 6.0 with built in consistency checks to control data output. Data were analyzed using Stata 13 for Windows. Categorical variables were summarized using proportions and continuous variables were presented as means with standard deviations or median with interquartile range. Descriptive analyses comprising computation of arithmetic means, frequencies and percentages were done on the study variables. Chi-square test was used to assess association between pneumococcal carriage and demographic characteristics. Bivariate and multiple logistic regression analyses were used to assess the relationship between pneumococcal carriage and other independent variables and results were presented as Odds Ratios (OR), *p* values and Confidence Intervals (95% CI). Statistical significance level was set at *p*-values less than 0.05. Permutations on vaccine serotype coverage of PCV-10, PCV-13 and PPV-23 on serotype distribution pattern were analyzed using the theoretical serotype coverage analytical method [36, 37].

### Ethical standards

Ethical approvals for this study were obtained from the KNUST Committee on Human Research Publication and Ethics (CHRPE/AP/371/18) and the Navrongo Health Research Centre Institutional Review Board (NHRCIRB314). The study was conducted in accordance with the regulatory requirements which affords greater protection to the subject under the human protection enactment stipulated in the declaration of Helsinki. All protocol methods were performed per the guidelines of the scientific and ethical review boards from which ethical approvals were sought.

## Results

### Demographic characteristics of children recruited into the study

193 participants aged between 5 and 12 years were enrolled into the study. The mean and median ages of participants were 8.6 (± 2.3) and 8 years (IQR 7–11) respectively. 54.4% were females (Table 1), and the majority (40.4%) of the participants were 8–10 years of age. Nearly all (98.4%) of the children had received their childhood immunization but only 24.4% (47) had received the PCV-13 vaccine. There were 37 children (19.2%) who had respiratory symptoms and 3 children (1.6%) who had been exposed to antibiotics prior to sampling. Participants from households bigger than the mean household size of seven were in the majority (56.5%, 109/193) (Table 1).

**Table 1:** Background Characteristics of the Study Participants

Variable	Number of children n (%)	Proportion with carriage n (%)	Unadjusted OR (95%CI)	P-value
Sex				
Male	88 (45.6)	32 (36.4)	1.36 (0.75–2.49)	0.313
Female	105 (54.4)	31 (29.5)		
Age (years)				
5—7	65 (33.7)	26 (40.0)	2.11 (0.93–4.78)	0.191
8—10	78 (40.4)	25 (32.1)	1.49 (0.67–3.34)	
11—12	50 (25.9)	12 (24.0)		
Childhood Immunization				
Yes	190 (98.4)	62 (32.6)		0.979
No	3 (1.6)	1 (33.3)	1.03 (0.09–11.6)	
Received PCV-13				
Yes	47 (24.4)	18 (38.3)	1.39 (0.7–2.76)	0.342
No	146 (75.6)	45 (30.8)		
Symptoms of RTI				
Yes	37 (19.2)	14 (37.8)	1.33 (0.63–2.8)	0.453
No	156 (80.8)	49 (31.4)		
Antibiotics taken				
Yes	3 (1.6)	1 (33.3)	1.03 (0.09–11.6)	0.979
No	190 (98.4)	62 (32.6)		
Household size				
< =7	109 (56.5)	37 (33.9)	0.56 (0.49–0.63)	0.660
> 7	84 (43.5)	26 (30.9)		
Total	**193**	**63 (32.6)**		

*Carriage proportions and p-values were calculated using the Chi-square test. Childhood immunizations vaccines; Bacillus Calmette-Guerin, Polio, Diphtheria, Tetanus, Pertussis, Hepatitis B, Pneumococcal, Rotavirus, Measles, Yellow fever and Men-A

### Carriage prevalence

The overall prevalence of *S. pneumoniae* among study participants was 32.6% (95%Cl, 26.0–40.0). Carriage rates of 40% (95% CI, 28.6–52.6), 32.1% (95% CI, 22.5–43.4) and 24.0% (95%CI, 13.9–38.2) were respectively recorded among age categories 5–7 years, 8–10 years and 11–12 years. Male and female participants recorded carriage rates of 36.4% (95% CI, 2–47) and 29.5% (95% CI, 22–39). Moreover, carriage prevalence of 38.3, 37.8 and 33.3% were observed for participants who were administered PCV-13, had respiratory symptoms and were exposed to antibiotics. Carriage prevalence for participants above and below the average household size was 33.9 and 30.9% respectively (Table 1).

In a sub-group analysis of pneumococcal carriage prevalence among 44 children who were eligible to receive PCV-13 when it was introduced in 2012, 40 children who were immunized against the pneumococcus had a carriage rate of 35% (95% CI, 21.0–52.0) compared to a carriage rate of 75% (95% CI, 4.0–100.0) among PCV-13 unvaccinated children.

### Pneumococcal serotype distribution and vaccine coverage

**Fig. 2:**
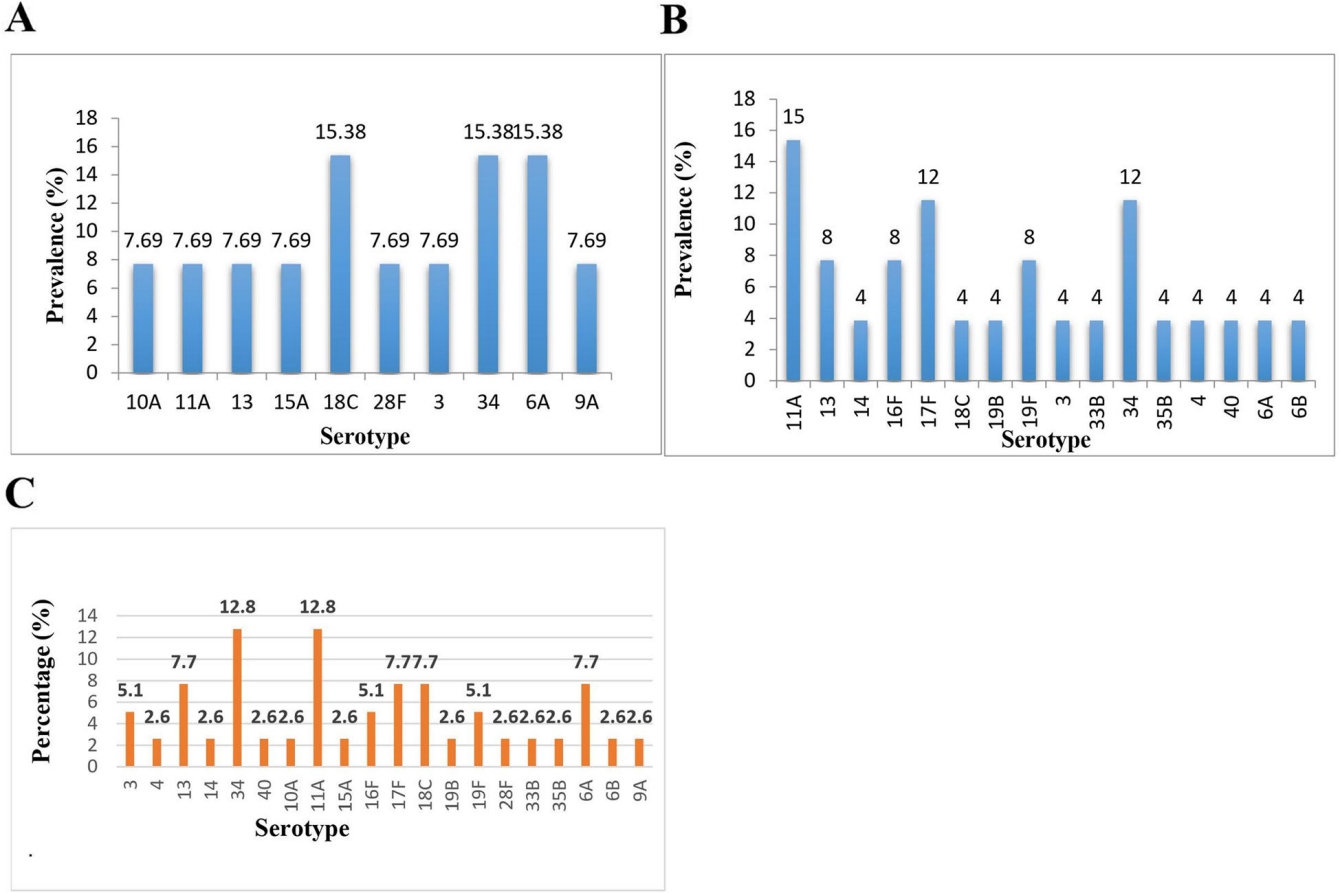
Serotype distribution of *S. pneumoniae* among children aged between 5 to 12 years. Serotypes are listed on the X-axis. **A** Serotype distribution among PCV-13 vaccinated children. **B** Serotype distribution among PCV-13 unvaccinated children. **C** Serotype distribution among all participants

Polysaccharide capsular typing analysis was carried out on 38 out of the 63 pneumococcal isolates. This was due to lack of viability of 25.4% (16/63) of the harvested isolates and 14.3% (9/63) being non-typeable. Nonetheless, twenty (20) distinct serotypes were identified; among which serotypes 35 and 11A were most predominant, each with a prevalence of 12.8% (5/39). Moreover, other prevailing serotypes recorded respective prevalence rates of 7.7% (13, 17F, 18C, 6A), 5.1% (16F, 3, 19F) and 2.6% (10A, 14, 15A, 19B, 28F, 33B, 35B, 4, 40, 6B, 9A). Only one participant carried multiple serotypes (1.6%) (Fig. 2).

Serotype distribution varied among PCV-13 vaccinated and PCV-13 unvaccinated children. Our results showed that the most commonly occurring serotypes among PCV-13 vaccinated children were serotypes 18C, 34, 6C, 11A, recording a uniform prevalence rate of 15.38% whereas serotypes 11(15%), 17F (15%) and 34 (12%) were predominant among PCV-13 unvaccinated group (Fig. 2).

**Fig. 3:**
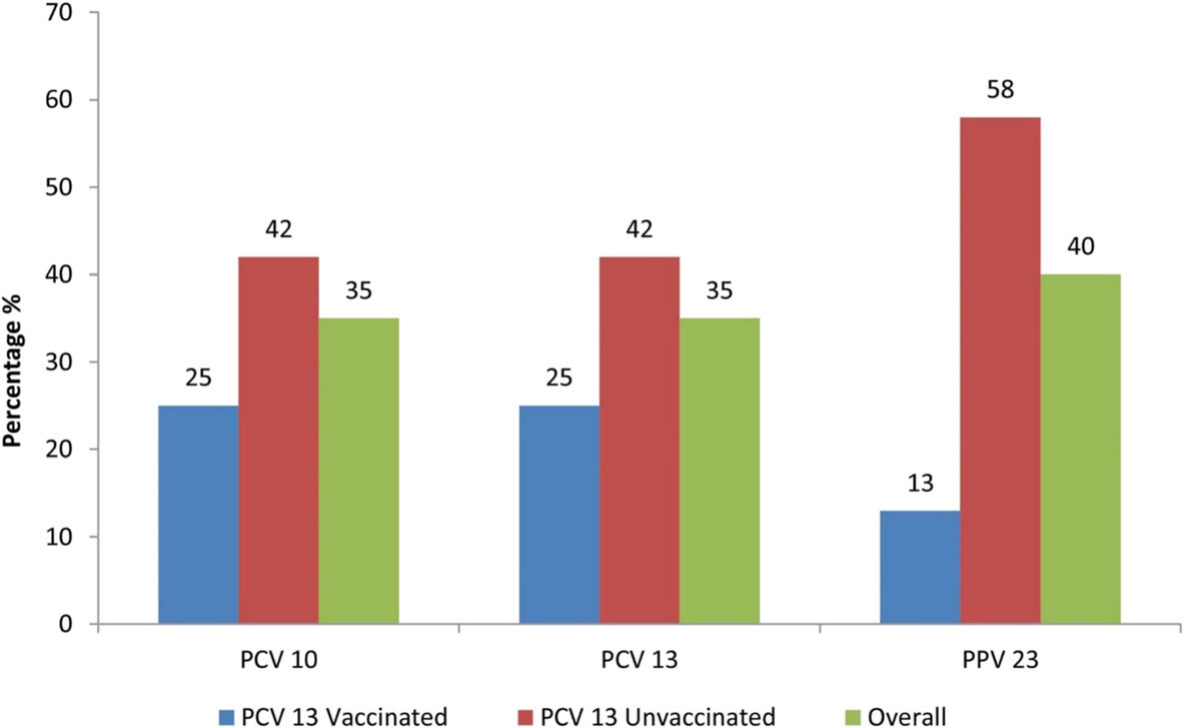
Comparison analysis of vaccine serotype coverage within the study population and among PCV-13 vaccinated and PCV-13 unvaccinated children. Vaccine targeted serotypes are present in lower proportions as indicated on the X-axis

A vaccine serotype coverage analysis showed a uniform serotype coverage rate 35% for all PCVs (PCV-10 and PCV-13) and a coverage rate of 40% for PPV 23. Additionally, a serotype coverage of 25% was observed for all PCVs among children vaccinated against the pneumococcus and a coverage of 42% was observed among PCV-13 unvaccinated children. Moreover, serotype coverage of 13 and 58% were observed among PCV-13 vaccinated and PCV-13 unvaccinated children respectively for PPV 23 (Fig. 3).

### Association between carriage and background factors

A bivariate and multivariate analysis performed to assess the association between carriage and other background factors (age, sex, PCV-13 vaccination, antibiotic exposure, respiratory symptoms and household size) did not show any statistically significant association between pneumococcal carriage and the background characteristics of the study participants (Table 2).

**Table 2:** Multivariate and Bivariate Risk Analysis Table

Variable	Proportion with carriage n (%)	Adjusted OR (95%CI)	Overall P-value
Sex			
Male	32 (36.4)	1.48 (0.80–2.75)	0.22
Female	31 (29.5)		
Age (years)			
5—7	26 (40.0)	2.26 (0.85–6.05)	0.10
8—10	25 (32.1)	1.58 (0.70–3.59)	
11—12	12 (24.0)		
Childhood Immunization			
Yes	62 (32.6)		0.99
No	1 (33.3)	0.95 (0.08–11.50)	
Received PCV			
Yes	18 (38.3)	0.95 (0.38–2.36)	0.91
No	45 (30.8)		
Symptoms of RTI			
Yes	14 (37.8)	1.26 (0.59–2.71)	0.55
No	49 (31.4)		
Antibiotics taken			
Yes	1 (33.3)	0.89 (0.08–10.30)	0.93
No	62 (32.6)		
Household size			
< =7	37 (33.9)		0.62
> 7	26 (30.9)	0.85 (0.46–1.60)	

*Odds ratios, confidence intervals and p-values were calculated using a bivariate and multivariate logistic regression analysis

## Discussion

The study investigated the nasopharyngeal carriage of *S. pneumoniae* among school children in the Kassena-Nankana districts of Northern Ghana. Though there was lack of association between pneumococcal carriage and background features, the rate of pneumococcal carriage, 32.6% (95%Cl, 26.0–40.0) observed among study participants was quite high. It was also observed that the majority (65%) of serotypes identified were non-vaccine type serotypes. Of notable concern was the observed high non-vaccine targeted strains in circulation. The persistently high rate of pneumococcal carriage despite various interventions implemented against the bacterium poses significant risk to the success of these interventions. The rate of pneumococcal carriage observed among the study participants conforms with findings of previous studies conducted pre and post implementation of PCV-13 which showed respective prevalence of 32% (95% CI 29–36%) to 39% (95% CI: 32.3 to 46.2) in 2013 and 2018 within the country [19, 36, 37]. However, carriage rates ranging between 27% (95% CI: 19.1 to 35.1) to 51% have been reported from other parts of the country, thus reflecting the variation in pneumococcal carriage prevalence per the different geographical locations [19, 23, 36, 37]. Relative to this study, a higher carriage rate of 54% (95% CI, 49–59%) was observed from a recent study post PCV-13 implementation in the country. This may be due to the lower age population (between 6 to 60 months) [23] which was investigated compared to the higher age group (5 to 12 years) in this study. Previous trends in carriage rates suggest that the pneumococcal carriage reservoir may be undergoing an epidemiological transition within the population. A pre-vaccination publication reported a higher rate of carriage in 2002 within the country followed by a drastic decline almost a decade after. Thus a carriage rate ranging from 51 to 32% was reported by the end of the pre-vaccination era [19, 38].

Recently published data on pneumococcal carriage prevalence post PCV-13 deployment within the country however showed no significant reduction in carriage rates among study participants. The study revealed a carriage rate of 54% (95% CI, 49–59%) among study participants from southern Ghana, reflecting the persistence in carriage despite vaccine deployments within the country [23].

Several countries have reported dramatic decline in the incidence of invasive pneumococcal disease (IPD) following implementation PCVs into their childhood immunization programme [39]. However, these immunization impacts are being supressed by the emergence of NVTs which are contributing to antimicrobial resistance developments translating in poor disease managements [12, 14].

In recent times carriage rates and disease incidents by VT have declined and a concurrent rise in in colonization by NVT post pneumococcal vaccination have become apparent globally [8, 40]. The 2016 meningitis outbreak in Ghana indicated that NVT 12F and 35 B were responsible for a proportion of cases reported during the outbreak season [41] whereas an increase in antibiotic-resistant NVTs, including serotype 19A, post-PCV 7 has been shown in other studies [13].

A comparison of PCV-13 serotype coverage among this study cohort showed a marked difference in prevalence between VT and NVT serotypes. A prevalence rate of 65% was observed for NVT serotypes relative to a prevalence rate of 35% for VT serotypes. In comparison with previous findings from Ghana in 2013, which reported a PCV-13 serotype coverage of 50%, the current study suggests a PCV-13 serotype coverage of 35% indicating a decline in PCV-13 serotype coverage. The observed drift in serotype distribution could be attributed to the replacement in serotypes by NVT serotypes which could be due to the effect of the currently used PCV-13 vaccine [42]. Reports from previous studies have suggested the role of genetic recombination in evolutions within the pneumococcal population including acquisition of antibiotic resistance and serotype-switching [43]. The upsurge in NVT within the pneumococcal reservoir with the increasing rates of antimicrobial resistance associated with these events [44] is of public health concern.

Earlier studies from Ghana and elsewhere in Africa have reported similar trends where a drastic decline in VT carriage was observed and the main attributable factor was vaccine driven [45]. Replacement serotypes may super evolve, evade herd immunity and drive NVT disease transmissions [46, 47].

We did not find any statistically significant association between carriage and the background characteristics of the children in the study. This could partly be attributed to the under power of the study, however, this study is important, in that it is the first of its kind to characterize the prevalence and serotype distribution of *S. pneumoniae* among carriers in the study area. This will serve as baseline data to guide future pneumococcal studies.

In this study, we could only serotype 38 (60.3%) out of the 63 isolates identified. This was because 16 (25.4%) were not viable and 9 (14.3%) were non-typeable. However, we compared the distribution of the background characteristics of the serotyped and non-serotyped groups, and found no statistically significant difference between them (Supplementary Table 1). Also, a prevalence rate of 15% [31] was used to estimate the sample size. However, subsequent studies have come out with varied prevalence, some of which are higher than the 15% prevalence that we used to estimate our sample size. We therefore recommend continuous surveillance to enhance comprehension on the dynamics of the pneumococcal carriage reservoir.

## Conclusion

Pneumococcal carriage persists within the populace despite the introduction of PCV-13 vaccine into Ghana’s EPI programme. Replacement in serotypes due to the limited serotype coverage of the current vaccine may be of minimal benefit to the populace. This therefore requires a review of the existing PCV vaccine to ensure optimal protection against the pathogen and wellbeing of the populace.

## Supplementary Information

**Additional file 1.**

**Additional file 2.**

## Data Availability

The datasets used for the study are available on request from the corresponding author (Deborah K. Narwortey, MPhil, Senior Research Scientist, Biomedical Department, Navrongo Health Research Centre, Email: narworteydeborah@gmail.com).

## Abbreviations

ATTC: American type culture collection
BHI: Brain heart infusion
MIC: Minimum inhibitory concentration
NHDSS: Navrongo Health and Demographic Surveillance System
NVT: Non-vaccine types
PCV-13: 13-Valent pneumococcal conjugate vaccine
VT: vaccine types
PPV: Pneumococcal polysaccharide vaccine
STGG: skim milk-tryptone-glucose-glycerin medium
