# Supplementary material for: Nasopharyngeal carriage of Streptococcus pneumoniae among healthy children in Kassena-Nankana districts of Northern Ghana
Source: BMC Infect Dis. 2021 Jul 8;21:661. doi: 10.1186/s12879-021-06302-5 (PMC8265090; doi:10.1186/s12879-021-06302-5)
Supplement: Supplementary file 1 — Additional file 1. [file 12879_2021_6302_MOESM1_ESM.docx]

**Nasopharyngeal Carriage of *Streptococcus pneumoniae* among Healthy Children in Kasena-Nankana Districts of Northern Ghana**

Deborah K. Narwortey^1,2*^, Alex O. Ofori^2^, Hans-Christian Slotved^3^, Eric S. Donkor^4^, Patrick O. Ansah^1^, Paul Welaga^1^, Godfred Agongo^1^, Abraham R. Oduro^1^

1. Navrongo Health Research Centre, Ghana.
2. Kwame Nkrumah University of Science and Technology, Kumasi, Ghana.
3. Department of Bacteria, Parasites and Fungi, Statens Serum Institut, Copenhagen, Denmark.
4. Department of Medical Microbiology, University of Ghana Medical School, Accra, Ghana.

***Corresponding author**

Deborah Korkor Narwortey

Navrongo Health Research Centre

Ghana Health Service

P.O. Box 114

Navrongo.

Email: [narworteydeborah@gmail.com](mailto:narworteydeborah@gmail.com)

AOO: owusu­_ofori@yahoo.com

HCS: [HCS@ssi.dk](mailto:HCS@ssi.dk)

ESD: ericsdon@hotmail.com

POA:[lonpoa@gmail.com](mailto:lonpoa@gmail.com)

PW: [pwelaga@yahoo.com](mailto:pwelaga@yahoo.com)

GA: [g_­agongo@yahoo.com](mailto:g_agongo@yahoo.com)

ARO: aroduro@gmail.com

**PARTICIPANT QUESTIONNAIRE**

| Participant ID: ………………  Name of School ………………………………………………. | Date of visit ……./……/…….  ^Day Month Year^ |
| --- | --- |
| 1. Informed consent obtained | Yes / No |
| 1. Do you prefer local language or English?   I. Local  II..English | Yes / No  Yes / No |
| 1. Sex | Male / Female |
| 1. Date of birth     **OR**  If date of birth is not known, Estimated Age | ……./……/……..  ^Day Month Year^ |
|  |  |
| 1. How many are you in your family? |  |
| 1. How many siblings do you have at home currently? |  |
| 1. Have you received the childhood immunization? | Yes / No |
| 1. If yes, have you received Pneumococcal vaccine? | Yes / No |
| 1. Are you experiencing signs and symptoms of any respiratory infections: such as cold, catarrh, cough, running nose, fever, etc? | Yes / No |
| 1. Have you taken any antibiotics within the past four weeks? | Yes / No |
