# Supplementary material for: Nasopharyngeal carriage of Streptococcus pneumoniae among healthy children in Kassena-Nankana districts of Northern Ghana
Source: BMC Infect Dis. 2021 Jul 8;21:661. doi: 10.1186/s12879-021-06302-5 (PMC8265090; doi:10.1186/s12879-021-06302-5)
Supplement: Supplementary file 2 — Additional file 2. [file 12879_2021_6302_MOESM2_ESM.docx]

**Nasopharyngeal Carriage of *Streptococcus pneumoniae* among Healthy Children in Kassena-Nankana Districts of Northern Ghana**

Deborah K. Narwortey^1,2*^, Alex O. Ofori^2^, Hans-Christian Slotved^3^, Eric S. Donkor^4^, Patrick O. Ansah^1^, Paul Welaga^1^, Godfred Agongo^1^, Abraham R. Oduro^1^

1. Navrongo Health Research Centre, Ghana.
2. Kwame Nkrumah University of Science and Technology, Kumasi, Ghana.
3. Department of Bacteria, Parasites and Fungi, Statens Serum Institut, Copenhagen, Denmark.
4. Department of Medical Microbiology, University of Ghana Medical School, Accra, Ghana.

***Corresponding author**

Deborah Korkor Narwortey

Navrongo Health Research Centre

Ghana Health Service

P.O. Box 114

Navrongo.

Email: [narworteydeborah@gmail.com](mailto:narworteydeborah@gmail.com)

AOO: owusu­_ofori@yahoo.com

HCS: [HCS@ssi.dk](mailto:HCS@ssi.dk)

ESD: ericsdon@hotmail.com

POA:[lonpoa@gmail.com](mailto:lonpoa@gmail.com)

PW: [pwelaga@yahoo.com](mailto:pwelaga@yahoo.com)

GA: [g_­agongo@yahoo.com](mailto:g_agongo@yahoo.com)

ARO: aroduro@gmail.com

**Supplementary Table 1: Comparison of The Background Characteristics Among Serotyped and Non-Serotyped Groups**

| **Variable** | **Serotyped**  **n (%)** | **Non-Serotyped**  **n (%)** | **P-Value** |
| --- | --- | --- | --- |
| **Sex** |  |  |  |
| Male | 19 (50.0) | 12 (48.0) | 0.877 |
| Female | 19 (50.0) | 13 (52.0) |  |
| **Age** |  |  |  |
| 5--7 | 14(36.8) | 12(48.0) | 0.58 |
| 8--10 | 17(44.8) | 8(32.0) |  |
| 11--12 | 7(18.4) | 5(20.0) |  |
| **Childhood Immunization** |  |  |  |
| Yes | 37(97.4) | 0(0.0) | 0.414 |
| No | 1 (2.6) | 25(100.0) |  |
| **Received PCV** |  |  |  |
| Yes | 13(34.2) | 5(20.0) | 0.222 |
| No | 25(65.8) | 20(80.0) |  |
| **Symptoms of RTI** |  |  |  |
| Yes | 8(21.1) | 6(24.0) | 0.783 |
| No | 30(78.9) | 19(76.0) |  |
| **Antibiotics taken** |  |  |  |
| Yes | 1 (2.6) | 0(0.0) | 0.414 |
| No | 37(97.4) | 25(100.0) |  |
| **Household size** |  |  |  |
| **<=7** | 24(63.2) | 13(52.0) |  |
| >7 | 14(36.8) | 12(48.0) | 0.379 |
| *****Serotype group: proportion of pneumococcal carriers whose specimens were serotyped. Non-serotype group: proportion of carriers whose specimen were not serotyped. | | | |
